# The Azithromycin Pro-Drug CSY5669 Boosts Bacterial Killing While Attenuating Lung Inflammation Associated with Pneumonia Caused by Methicillin-Resistant Staphylococcus aureus

**DOI:** 10.1128/aac.02298-21

**Published:** 2022-08-16

**Authors:** Anno Saris, Wanhai Qin, Christine C. A. van Linge, Tom D. Y. Reijnders, Sandrine Florquin, Michael Burnet, Simon Strass, Alex F. de Vos, Tom van der Poll

**Affiliations:** a Center for Experimental and Molecular Medicine, Amsterdam University Medical Centers, Academic Medical Center, University of Amsterdam, Amsterdam, the Netherlands; b Department of Infectious Diseases, Leiden University Medical Center, Leiden, the Netherlands; c Department of Pathology, Amsterdam University Medical Centers, Academic Medical Center, University of Amsterdam, Amsterdam, the Netherlands; d Synovo GmbH, Tübingen, Germany; e Department of Pharmaceutical Sciences, Eberhard-Karls-University Tübingen, Tübingen, Germany; f Department of Infectious Diseases, Amsterdam University Medical Centers, Academic Medical Center, University of Amsterdam, Amsterdam, the Netherlands

**Keywords:** drug resistance, host directed therapies, MRSA, Staphylococcus aureus, host-pathogen interactions, immune response

## Abstract

Antibiotic resistance is a major problem, with methicillin-resistant Staphylococcus aureus (MRSA) being a prototypical example in surgical and community-acquired infections. S. aureus, like many pathogens, is immune evasive and able to multiply within host immune cells. Consequently, compounds that aid host immunity (e.g., by stimulating the host-mediated killing of pathogens) are appealing alternatives or adjuncts to classical antibiotics. Azithromycin is both an antibacterial and an immunomodulatory drug that accumulates in immune cells. We set out to improve the immunomodulatory properties of azithromycin by coupling the immune activators, nitric oxide and acetate, to its core structure. This new compound, designated CSY5669, enhanced the intracellular killing of MRSA by 45% ± 20% in monocyte-derived macrophages and by 55% ± 15% in peripheral blood leukocytes, compared with untreated controls. CSY5669-treated peripheral blood leukocytes produced fewer proinflammatory cytokines, while in both monocyte-derived macrophages and peripheral blood leukocytes, phagocytosis, ROS production, and degranulation were unaffected. In mice with MRSA pneumonia, CSY5669 treatment reduced inflammation, lung pathology and vascular leakage with doses as low as 0.01 μmol/kg p.o. CSY5669 had diminished direct *in vitro* antibacterial properties compared with azithromycin. Also, CSY5669 was immunomodulatory at concentrations well below 1% of the minimum inhibitory concentration, which would minimize selection for macrolide-resistant bacteria if it were to be used as a host-directed therapy. This study highlights the potential of CSY5669 as a possible adjunctive therapy in pneumonia caused by MRSA, as CSY5669 could enhance bacterial eradication while simultaneously limiting inflammation-associated pathology.

## INTRODUCTION

Pneumonia is a major cause of morbidity and mortality (1) and is the most common infectious source in patients with sepsis (2). In 2019, lower respiratory tract infections affected an estimated 489 million people globally and were considered responsible for over 2.5 million deaths (3). Methicillin-resistant Staphylococcus aureus (S. aureus) (MRSA) is a leading causative microorganism in nosocomial pneumonia and is an emerging pathogen in community-acquired pneumonia (4, 5). MRSA pneumonia can be associated with extensive inflammation as well as concomitant damage and necrosis of lung tissue (4, 5). Thus, the modification of this harmful host response may be an attractive target for adjunctive therapy in patients with this infection.

Macrolides, characterized by their macrolactone ring, inhibit bacterial protein synthesis by binding to the 50S subunit of a bacterial ribosome (6). While bacteria can readily acquire resistance, macrolides are molecules that are capable of modulating immune responses, and they are used to do so in chronic respiratory diseases, such as cystic fibrosis or diffuse panbronchiolitis (6, 7). The immunomodulatory effects of macrolides are complex, diverse, and context-dependent (described in detail elsewhere) (6); in general, in the absence of (over)inflammation, macrolides seem to promote immune functions while also preventing the overactivation of the immune system and promoting the repair of inflammation-induced tissue damage during infections. However, the use of azithromycin (AZM) at the usual dosage (250 mg) as an immunomodulatory therapy is controversial, given the potential to continually select for antimicrobial resistance (8). Thus, eliminating its bacteriostasis in the immune modulatory mode and/or improving its immumodulating potency is desirable. In order to do so, macrolides can be chemically modified to allow for the linkage of specific side groups that may boost a specific host’s immune function (9, 10).

Simply boosting the antibacterial immune response can be mediated by a vast number of molecules (e.g., bacterial by-products and TLR ligands), but ideally, these molecules should be self-limiting (i.e., should also regulate the immune balance). Nitric oxide (NO) has an important regulatory role during infections and is involved in sepsis pathophysiology. Being a reactive nitrogen species, NO directly impairs bacterial survival and additionally stimulates proinflammatory responses while also depleting nutrients that may be exploited for bacterial growth (11). However, high systemic levels of NO impair host defense due to vascular leakage and the inhibition of neutrophil influx to the site of infection (11–13). These side effects may be eliminated by specifically delivering NO to inflammatory foci, rather than relying on systemic administration, while keeping the advantageous effects of NO. Alternatively, short-chain fatty acids (SCFAs), comprising acetate, butyrate, and propionate, are end products of bacterial fermentation that have been hypothesized to correct an imbalanced immune response (14, 15). SCFAs can either stimulate or inhibit immunity, depending on environmental cues (16). During pathogen invasion, SCFAs can be released systemically by innate cells to stimulate the systemic release of interferon-γ, which facilitates host resistance against bacterial infection (17, 18).

In the current study, we optimized the immunomodulatory capacities of AZM and minimized its *in vitro* bacteriostatic properties. The new compound CSY5669 contains a 4′′ nitrate-releasing ester which induces NO (by mechanisms described previously) (19) and a 2′ acetate on an AZM backbone. Here, we set out to investigate the effects of CSY5669 *in vitro* and in an acute infection of the airway by MRSA as well as to identify its underlying mechanisms of action.

## RESULTS

### Chemical structure of CSY5669.

**FIG 1:**
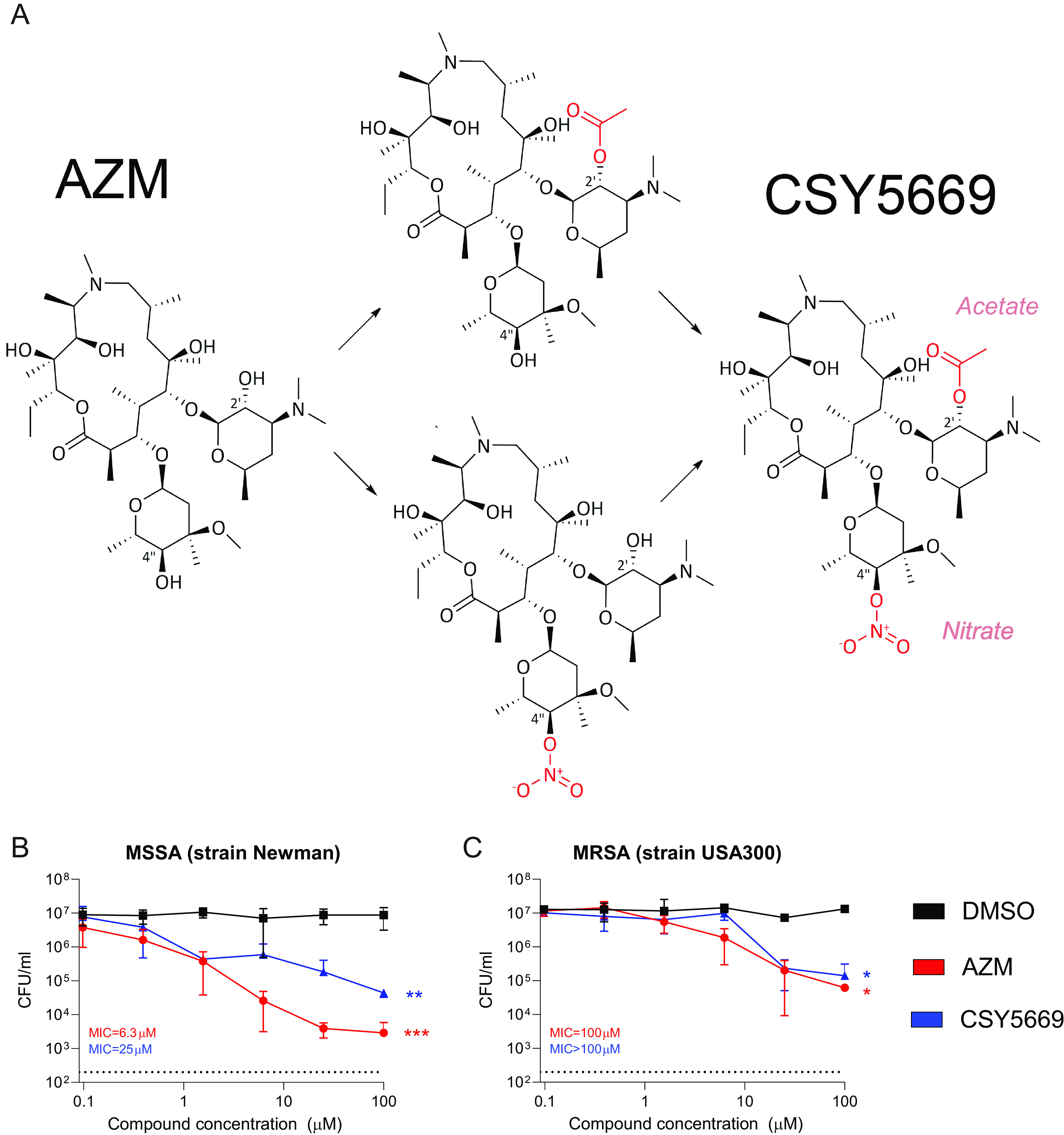
Modulation of azithromycin to produce CSY5669 with limited antibiotic activity. Azithromycin (AZM) was chemically modified to allow for linkage with an acetate and a nitrate ester (NO donating group), wherein acetate invariably reacted at the 2′ position of the desosamine, followed by the 11-position, while nitrate esters were formed preferentially at the 4′′ position. Both new functional side groups are depicted in red (A). The antibiotic activity of AZM and CSY5669 was determined by incubating methicillin-susceptible and methicillin-resistant Staphylococcus aureus (MSSA [B] and MRSA [C], respectively) in broth with increasing concentrations of azithromycin (red), CSY5669 (blue) or DMSO (0.001 to 1%; black), *n* = 2. Bacterial concentration was determined after 2 h incubation, and minimum inhibitory concentrations were established after overnight incubation. The dotted line represents the lower limit of detection. Statistical significance was tested using one-way ANOVA with Tukey's *post hoc* test. *, *P* < 0.05; **, *P* < 0.01; ***, *P* < 0.001.

AZM possesses 5 hydroxy groups, of which two, at the 2′ and 4′′ positions, were substituted to carry additional functional groups. To enhance the immunomodulatory capacity, the low molecular weight, danger-associated molecular pattern NO and the SFCA acetate were coupled to AZM. Steric effects, anhydrate reaction conditions, and vicinal atoms dictate the order of reactivity of the groups, and this has the effect that acetates invariably react at the 2′ position of the desosamine, followed by the 11-position, while nitrate esters are formed preferentially at the 4′′ position (Fig. 1) to form CSY5669.

The 2′ hydroxy in desosamine is essential for the binding of AZM to bacterial ribosomes. This part of the molecule is blocked by an acetate ester in CSY5669, so we hypothesized that CSY5669 has reduced bacteriostatic effects. At high concentration, both AZM and CSY5669 inhibit the growth of both methicillin-susceptible S. aureus (MSSA, Newman strain) and MRSA (USA300/BK11540 strain) in liquid cultures. In a classic minimum inhibitory concentration (MIC) assay that incubates bacteria overnight in broth, CSY5669 also appeared less bacteriostatic than AZM: 100 μM versus 6.3 μM for MSSA and >100μM versus 100 μM for MRSA (Fig. 1B and C).

### CSY5669 inhibits lung inflammation and concomitant tissue damage during murine MRSA pneumonia.

**FIG 2:**
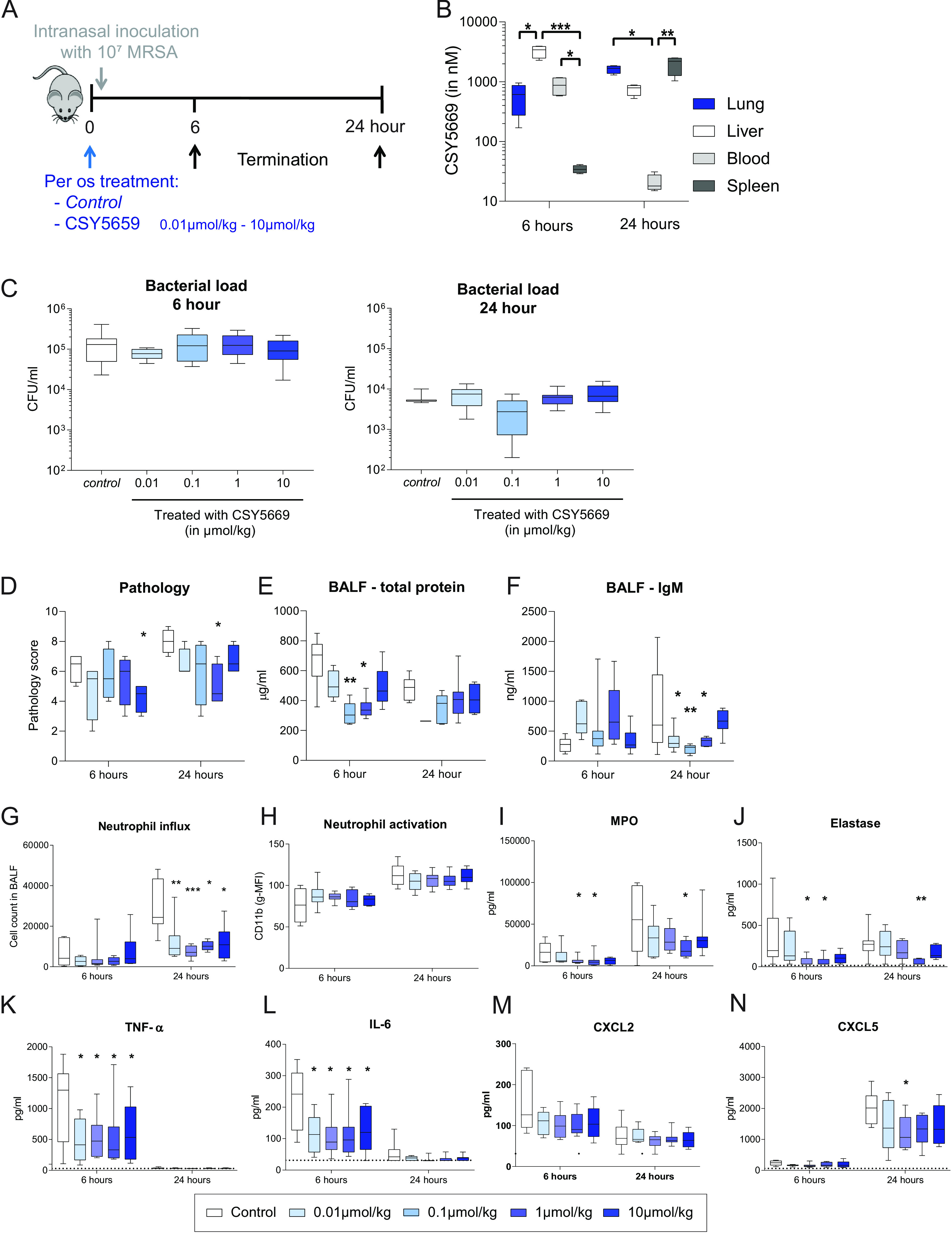
CSY5669 reduces lung inflammation and protein leakage during murine MRSA pneumonia. C57BL/6J mice were treated with 0 to 10 μmol/kg CSY5669 and were, directly thereafter, intranasally instilled with 10^7^ CFU MRSA ([A], *n* = 8 per group). After 6 h or 24 h, the mice were sacrificed to determine the pharmacokinetics of CSY5669 ([B], *n* = 4), lung bacterial load ([C], *n* = 8) or lung damage ([D], *n* = 4) in extracted organs after homogenization (B and C) or formalin fixation (D). Parameters of vascular leakage (total protein and IgM) were determined in bronchoalveolar lavage fluid (BALF) obtained after one-sided BAL ([E, F], *n* = 4 to 8, except 24 h 0.01 mmol/kg CSY5669, for which *n* = 1). Cells were isolated from BALF using centrifugation, after which cells were phenotyped and enumerated using flow cytometry ([G, H], *n* = 6 to 8 per group; for the gating strategy used, see Fig. S1 in the supplemental material). Concentrations of myeloperoxidase ([I]; MPO), elastase (J), TNF-a (K), IL-6 (L), CXCL2 (M) and CXCL5 (N) were determined in supernatant BALF using ELISA. Data are presented as median 6 interquartile range (box) 6 range (whiskers). Differences from controls were tested for statistical significance using the Kruskal-Wallis test with Dunn’s *post hoc* test (B to E). *, *P* < 0.05; **, *P* < 0.01; ***, *P* < 0.001.

To investigate whether CSY5669 is a potential therapeutic in MRSA pneumonia, mice were treated with either 0.01 to 10 μmol/kg CSY5669 or vehicle via oral gavage directly after the intranasal instillation of 10^7^ MRSA (see Fig. 2A for the experimental setup). Six hours after administration, CSY5669 concentrations were highest in the liver (median 3,424 nM in the 10 μmol/kg treatment group) and were also detectable in the lung (median 610 nM in the 10 μmol/kg treatment group) and the blood (median 874 nM in the 10 μmol/kg treatment group) (Fig. 2B). At 24 h postadministration, lung and spleen concentrations had risen (median 1,675 nM and 2,207mnM in the 10 μmol/kg treatment group), while liver concentrations had declined (median 792 nM in the 10 μmol/kg treatment group). Treatment with lower concentrations had similar pharmacokinetics to linear dose effects in terms of organ concentrations. Important in the context of this study is that even at the highest dose, compound concentrations were well below the MIC for the MRSA strain used in this study.

As previously described (20, 21), mice were capable of clearing the infection in the absence of treatment with a median bacterial load of 1.31 × 10^5^ CFU/mL (range: 2.3 × 10^4^ CFU/mL to 4.1 × 10^5^ CFU/mL) 6 h after infection, which further reduced to 5.0 × 10^3^ CFU/mL (range: 4.6 × 10^3^ CFU/mL to 1.0 × 10^4^ CFU/mL) 24 h after infection. Bacterial levels were not affected by treatment with any of the four concentrations of CSY5669 (Fig. 2C). However, the extent of inflammation-associated lung pathology (quantified as listed in the Methods section) was reduced after CSY5669 treatment, without clear differences between treatment concentrations (Fig. 2D). CSY5669 treatment also reduced vascular leakage 24 h, but not 6 h, after infection, as measured by total protein and IgM concentration in bronchoalveolar lavage fluid (BALF) (Fig. 2E).

To gain insight into the effect of CSY5669 on inflammatory responses in the lung during MRSA pneumonia, we investigated cell influx and activation as well as cytokine and chemokine release in BALF. BALF contained predominantly alveolar macrophages and neutrophils (data not shown), wherein neutrophil numbers increased up to 24 h postinfection (Fig. 2G), which associated with the enhanced activation of infiltrated cells, as indicated by enhanced surface CD11b expression (Fig. 2H) and elevated levels of the neutrophil degranulation products: myeloperoxidase (MPO) and elastase (Fig. 2I and J). CSY5669 treatment reduced neutrophil influx by approximately 50% at 24 h, at all doses from 0.01 μmol/kg, without a clear effect of dosage. While CSY5669 did not impact neutrophil CD11b expression, it did reduce MPO and elastase levels in BALF, possibly reflecting the reduced neutrophil numbers in BALF. MRSA pneumonia was associated with high concentrations in BALF of proinflammatory cytokines (TNF-α, IL-6) (Fig. 2K and L) and chemokines (CXCL2, CXCL5) (Fig. 2M and N). CSY5669 treatment at all doses profoundly reduced BALF levels of TNF-α and IL-6 (at 6 h) and modestly diminished BALF CXCL5 levels (at 24 h). To determine whether CSY5669 directly affects neutrophil migration (and thereby, to explain the observed reduced neutrophil influx), a chemotaxis assay was performed using isolated human neutrophils. While neutrophils readily migrated toward IL-8 and fMLP, CSY5669 did not affect this migration (Fig. S4).

**FIG 3:**
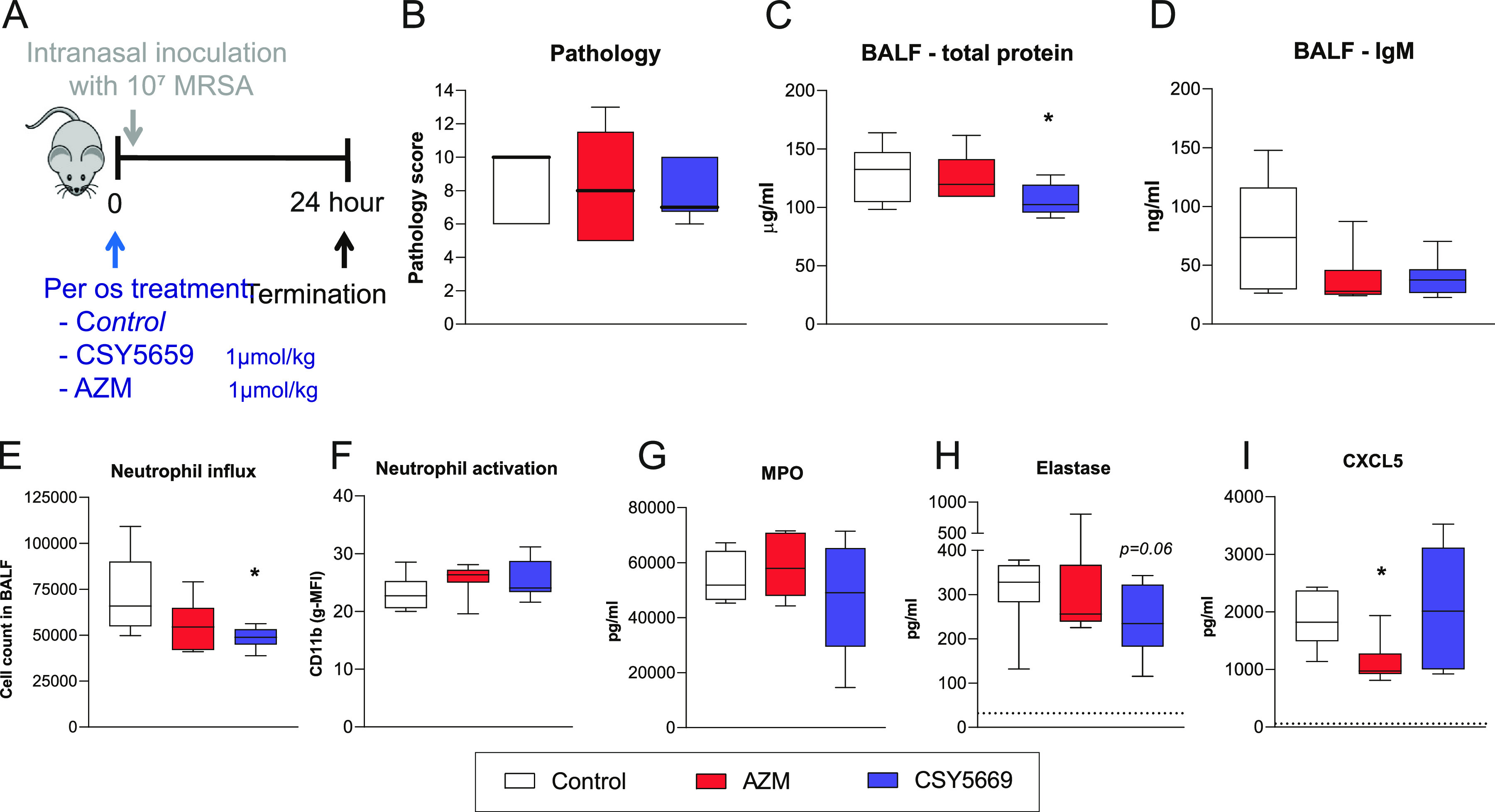
CSY5669 inhibits neutrophil recruitment and cytokine release during murine MRSA pneumonia more potently as AZM. MRSA pneumonia was induced in mice using the model presented in Fig. 3A, wherein BALF was obtained using one-sided BAL. Lung damage ([B], *n* = 4) was quantified in formalin fixed extracted organs. Parameters of vascular leakage (total protein and IgM) were determined in bronchoalveolar lavage fluid (BALF) obtained after one-sided BAL [C, D]. Cells were isolated from BALF using centrifugation, after which cells were phenotyped and enumerated using flow cytometry ([E, F]; for gating strategy, see Fig. S1 in the supplemental material). Concentrations of myeloperoxidase ([G]; MPO), elastase (H), and CXCL5 (I) were determined in supernatant BALF using ELISA. Dotted lines represent lower limits of detection. Data are presented as median ± interquartile range (box) ± range (whiskers). Differences from controls were tested for statistical significance using the Kruskal-Wallis test with Dunn’s *post hoc* test. *, *P* < 0.05; **, *P* < 0.01; ***, *P* < 0.001.

To investigate whether CSY5669 had improved immunomodulatory capacities compared to its AZM-backbone, mice were treated with either CSY5669 or AZM after intranasal MRSA installation (see Fig. 3A for the experimental setup). From the above dose-escalation study, 1 μM was selected as the most efficacious for CSY5669, and the same dosage was used for AZM. Again, no effects on bacterial loads were observed after either treatment (data not shown), but tissue pathology and vascular leakage (as determined by total protein and IgM) were reduced after AZM treatment and CSY5669 treatment, which was less pronounced (and not significant) (Fig. 3B to D). Furthermore, CSY5669 significantly impaired neutrophil influx, with a trend in reduced MPO and elastase levels, none of which were significantly affected by AZM (Fig. 3E to H). AZM did significantly reduce chemokine levels, while CSY5669 did not (Fig. 3I). In conclusion, some of the effects observed after CSY5669 treatment seem to be attributable to the AZM-backbone, but CSY5669 modulated the immune response more potently than did AZM.

### CSY5669 enhances the intracellular killing of MRSA by human macrophages and blood leukocytes.

**FIG 4:**
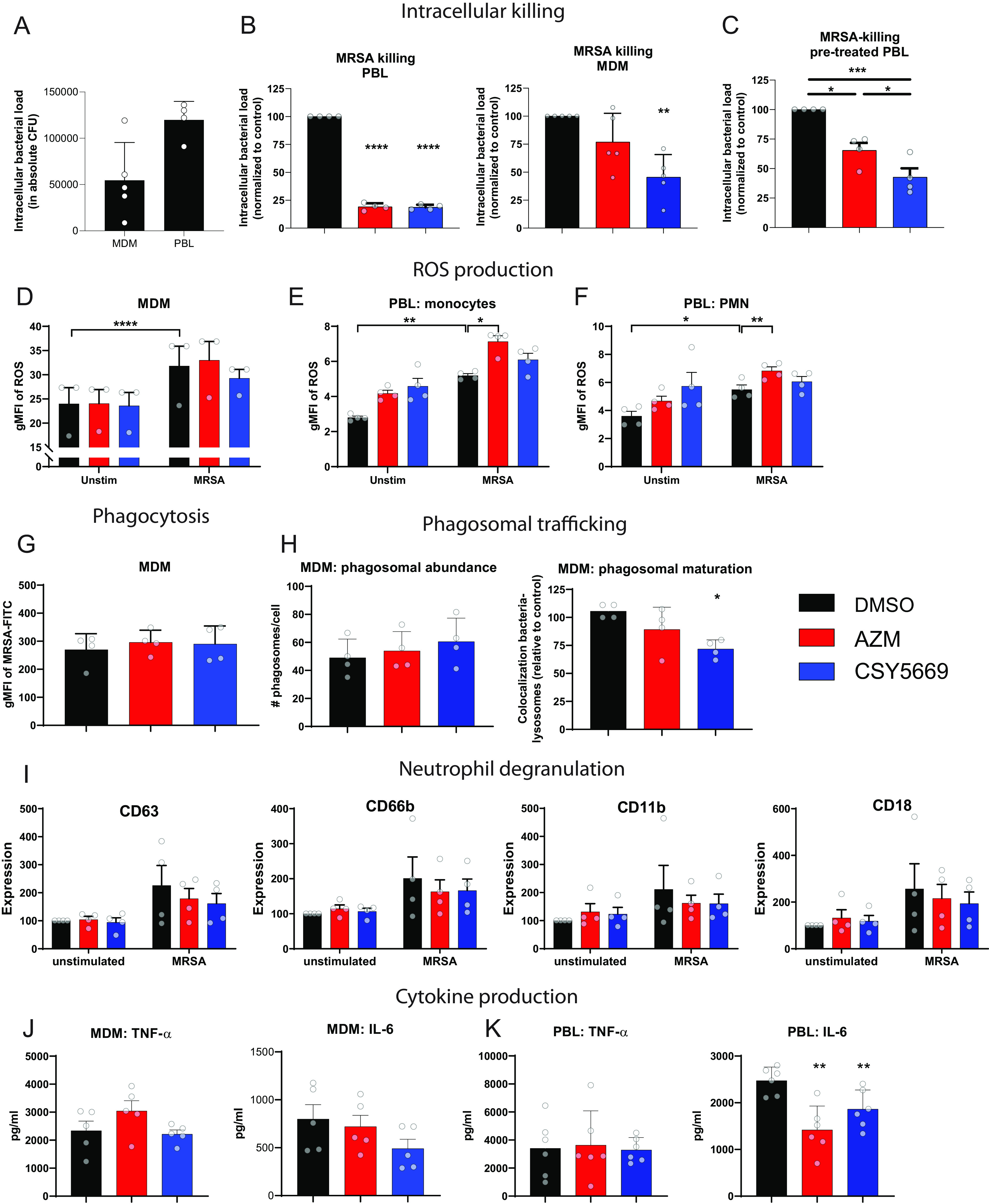
Effect of CSY5669 on antimicrobial and inflammatory responses by monocyte-derived macrophages and blood leukocytes. Monocyte-derived macrophages (MDMs, *n* = 5) or peripheral blood leukocytes (PBL, *n* = 4) were infected with MRSA and subsequently incubated overnight (MDMs) or 6 h (PBL) in the presence of 1 μM AZM or CSY5669, after which intracellular bacterial numbers were determined (A) and presented, relative to DMSO controls (B). Alternatively, PBLs were pretreated with AZM or CSY5669, washed, and subsequently infected and incubated with MRSA for 6 h (C). MDMs ([D], *n* = 3) or PBLs (gated on viable CD14^+^ monocytes [E] or on viable CD66b^+^ neutrophils [F], *n* = 4; for the gating strategy used, see Fig. S3 in the supplemental material) were incubated with heat-killed MRSA for 60 min in the presence of ROS-reactive dye, after which cells were measured with flow cytometry. Monocyte-derived macrophages (MDMs) were incubated with heat-killed, FITC-labeled MRSA for 30 min and measured with flow cytometry ([G], *n* = 4). MDMs were infected with viable MRSA and incubated for 120 min, after which cells were fixed and measured using confocal microscopy to determine the phagosomal abundance (number of lysotracker^+^ organelles per cell) or the colocalization of phagolysosomes with bacteria ([H], *n* = 4). Isolated neutrophils were incubated for 60 min with heat-killed MRSA, after which the expression of degranulation markers (CD63: azurophilic/primary granules, CD66b: specific/secondary and tertiary granules, CD11b: secretory vesicles, CD18: general activation marker) was determined ([I], *n* = 4). Expression levels were normalized to unstimulated DMSO-treated controls. MRSA-induced cytokine release was determined in MDMs ([J], overnight incubation, *n* = 5) and PBLs ([K], 4 h incubation, *n* = 6). Data are presented as mean ± standard deviation, with each dot representing (the average of) individual experiments. Statistical significance was tested using a one-way analysis of variance with Tukey’s *post hoc* test. *, *P* < 0.05; **, *P* < 0.01; ***, *P* < 0.001; ****, *P* < 0.0001.

To assess whether CSY5669 promotes the elimination of intracellular bacteria, human monocyte-derived macrophages (MDMs) and peripheral blood leukocytes (PBLs) were infected with MRSA, after which the intracellular killing of MRSA was monitored in the presence of CSY5669 or AZM. Cells were treated with 1 μM CSY5669, which does not affect cellular viability (data not shown) and excludes any direct antibacterial effects, given the >100μM MIC of CSY5669 for MRSA. In MDMs, CSY5669 reduced intracellular bacterial loads by 45% ± 20% (mean ± standard deviation) compared with the control (*P* = 0.008), while AZM did not significantly reduce intracellular bacterial loads (23% ± 26%, *P* = 0.22 versus the control) (Fig. 4B). In PBLs, CSY5669 and AZM reduced bacterial loads to a similar degree (82% ± 2% and 82% ± 3% relative to the control) (Fig. 4B). As there is some concern that macrolides may have increased antibacterial activity in specific cell culture media (22), and moreover, to investigate whether PBLs are reprogrammed by CSY5669 treatment, cells were pretreated with either AZM or CSY5669 and only incubated with bacteria after the treatment had been removed. With this experimental setup, both CSY5669 and AZM significantly reduced intracellular bacterial loads compared with controls, with CSY5669 showing a significantly greater effect than that of AZM (55% ± 15% and 29% ± 22%, respectively) (Fig. 4C). Collectively, these results show that while CSY5669 has a limited direct antimicrobial activity against MRSA, it especially enhances the intracellular killing of MRSA by MDMs and PBLs.

Immune cells have many strategies by which to eradicate pathogens, and alterations to any of these could contribute to the observed enhanced intracellular killing. One important effector mechanism for the eradication of intracellular bacteria is the production of reactive oxygen species (ROS) (23). In MDMs, ROS production was unaffected by either treatment (Fig. 4D). However, in PBLs, AZM treatment, but not CSY5669 treatment, enhanced MRSA-induced ROS production (*P* = 0.02 and *P* = 0.006 versus control in monocytes and neutrophils that were gated separately, respectively) (Fig. 4E and F). Spontaneous ROS production, which ultimately leads to oxidative stress, was not affected by either AZM or CSY5669 in either MDMs or PBLs (Fig. 4D to F).

MDMs are excellent at degrading cargo via the phagolysosomal system, wherein a pivotal first step is phagocytosis (23). To investigate the effect of CSY5669 on phagocytosis, MDMs were treated with 1 μM CSY5669 or AZM and incubated with heat-killed, FITC-labeled MRSA. Neither CSY5669 nor AZM affected phagocytosis (Fig. 4G). After being phagocytosed, bacteria will be encapsulated into phagosomes that slowly mature and ultimately fuse with lysosomes. To investigate whether CSY5669 affects the phagolysosomal degradation system of MDMs, MRSA-infected MDMs were imaged using Lysotracker, a stain that is specific for late phagosomes and phagolysosomes. CSY5669 did not affect the total number of phagosomes but surprisingly reduced the colocalization of MRSA with phagolysosomes, while AZM had no effect (Fig. 4H).

Neutrophils express many pre-produced antimicrobial peptides that can be rapidly secreted upon pathogen encounter (23). The release of neutrophils’ antimicrobial peptides can be monitored by measuring surface-expressed CD63, CD66b, or CD11b for primary granules, secondary/tertiary granules, and secretory vesicles, respectively (24). Stimulating neutrophils with MRSA induced neutrophil activation, as measured by CD18, and degranulation of all three granules (Fig. 4I). Neither CSY5669 nor AZM affected neutrophil activation or degranulation *in vitro*.

The production of TNF-α and IL-6 by macrophages was not significantly affected by CSY5669 (Fig. 4J). In PBLs, TNF-α production was also unaffected, but MRSA-induced IL-6 production was significantly reduced by both AZM and CSY5669 (Fig. 4K).

## DISCUSSION

Over the last decade, bacterial antibiotic resistance has developed at an alarming rate due to the overuse and abuse of antibiotics, with one of the most prototypical examples of resistant bacteria being MRSA. Host directed therapies that stimulate immune cells to clear an infection and/or limit inflammation-associated pathology could be an alternative or adjunct to antibiotic therapy, as host directed therapies bypass existing resistance mechanisms and potentially spare the commensal microbiome from nonspecific effects, as observed in classical antibiotic therapies. By modifying AZM, we developed a novel macrolide, CSY5669, which is capable of releasing two innate signaling molecules, NO and acetate, with excellent targeting to the site of infection, likely due to the ability of macrolides to accumulate in phagocytes. Here, we show that, compared to AZM, CSY5669 more potently increases the intracellular killing of MRSA by immune cells *in vitro* in the absence of a direct antimicrobial effect and also attenuates characteristic features of lung inflammation and concomitant tissue pathology during acute MRSA pneumonia *in vivo*.

One of the most striking results obtained *in vivo* was that CSY5669 treatment at 0.01 μmol/kg halved the number of neutrophils recruited to clear the infection. Treating mice with CSY5669 during pneumonia reduced MRSA inflammation-induced tissue damage and vascular leakage, which would be beneficial for patients with MRSA pneumonia, who frequently suffer severe inflammation-associated lung injury (4, 5), as well as potentially for many other airway infections that involve inflammation-associated lung injuries that are mediated by proteases released from neutrophils. The reduced tissue pathology after CSY5669 treatment could be due to the neutrophil influx, which is reduced by half while maintaining bacterial killing. Despite there being fewer neutrophils, the infection was cleared at least as fast in the CSY5669 groups as in untreated mice, and this was accomplished with less overall tissue damage. The ability to clear more bacteria with fewer cells is a phenomenon that we term “neutrophil efficiency”. Important in this concept is that there is a gain of neutrophil function, as opposed to a simple inhibition of their infiltration (l.oss of function). The observation that the rate of clearance was similar with half as many immune cells is consistent with the 50% increase in killing that was observed in the *in vitro* assays.

While CSY5669 enhanced intracellular killing by human MDMs and PBLs, the mechanism of action remains elusive. Previous studies reported the capacity of macrolides to increase the intraphagosomal killing of Aggregatibacter actinomycetemcomitans (25), Streptococcus pyogenes (26), and Candida albicans (27) by currently unidentified mechanisms. In line with this, AZM also enhanced the intracellular killing of MRSA, albeit to a lesser extent than did CSY5669, suggesting that additional mechanisms are at play in the latter. Immune cells can exploit various strategies by which to kill bacteria, wherein the first pivotal step for intracellular killing is phagocytosis, which was not affected by CSY5669 (or AZM) *in vitro*. Macrolides have been reported to affect phagocytosis, but these reports are inconsistent, with both enhanced and reduced phagocytosis being reported (26, 28, 29). After phagocytosis, bacteria are directed to early phagosomes, which progressively acidify to ultimately fuse with lysosomes. However, AZM inhibits the acidification of (auto)phagosomes, and this may be a general characteristic of macrolides, which are organic bases (30, 31). In line with this, CSY5669 reduced the colocalization of bacteria with acidic organelles in MDMs. Preventing bacterial transfer to the phagolysosomes may provide an insight into the mode of action of CSY5669. The natural cycle of S. aureus infections normally involves the invasion and lysis of phagocytes. Intracellular growth of MRSA occurs predominantly in acidic phagosomes (32, 33), and, for most strains, acidification seems to be necessary for their survival within host cells (33–35), presumably because it induces the expression of virulence factors (34). Thus, by inhibiting (certain aspects of) the transit from phagosomes to phagolysosomes, which prevents S. aureus from occupying its natural niche, CSY5669 may actually serve to restrict the intracellular growth of S. aureus, rather than to enhance its killing.

S. aureus presumably escapes intraphagosomal destruction within host cells by weakening and subsequently disrupting vacuolar membranes via the secretion of Staphylococcus-secreted, pore-forming toxins combined with mechanical stress due to bacterial replication (33). AZM is reported to reduce lysosomal oxidative stress and concomitant lysosome leakage (36), which may thereby limit the lysosomal escape of S. aureus. To facilitate its intracellular survival, S. aureus impairs the activity of many host bactericidal proteins (37), while AZM is shown to enhance protein degradation (31), likely by enhancing the activity of certain lysosomal enzymes (38). These studies, however, must be interpreted with caution, as data from high-dose *in vitro* studies may not always translate to low dose *in vivo* studies. It is not yet clear whether CSY5669 shares these effects or whether the signaling by its side-groups induces more specific bacterial killing processes.

The immunomodulatory effects of CSY5669 during murine MRSA pneumonia may be partially attributable to the AZM backbone and/or the esterified signal molecules, NO and acetate. Addressing the macrolide hypothesis first: in mice with pneumonia caused by macrolide-resistant Streptococcus pneumoniae, AZM (100 mg/kg), administered in combination with ceftriaxone decreased the neutrophil influx in BALF (39). In a ventilator-associated pneumonia mouse model caused by Acinetobacter baumannii, AZM (10 or 100 mg/kg) reduced the neutrophil influx and the release of IL-1β, IL-6, and CXCL2 in BALF (40). Consistent with this, patients with community-acquired pneumonia who were treated with macrolide containing regimens (details not stated, but typically AZM 3.5 to 7 mg/kg; 250 or 500 mg/day) had lower levels of TNF-α and IL-6 in BALF compared with patients treated with nonmacrolide regimens (41). These data show that while at high doses (i.e., above ~5mg/kg/day or 250 mg/day), macrolides, AZM in particular, can modulate inflammation and neutrophil influx without affecting bacterial loads, though CSY5669 is more potent and specific, as its immunomodulating effects have already been observed with doses as low as 0.01 to 0.1 μmol/kg (i.e., 0.008 to 0.08 mg/kg).

In contrast to the AZM backbone, CSY5669 “donates” an NO as well as an acetate molecule, both of which are immunomodulatory. NO, a pleiotropic molecule with many downstream effects, has been shown to facilitate host survival during S. aureus infections without affecting bacterial loads (42), exactly in line with our observations. The host-beneficial effects of NO during infection may involve its effector function as a reactive nitrogen species, its induction of iNOS, or its simple limitation of leukocyte recruitment by downregulating chemokines and adhesion molecules on endothelial cells (11, 42). Acetate, like NO, has many potential effects on the immune system, and these effects are highly context-dependent (14, 15). Acetate can limit the recruitment and degranulation of neutrophils, both of which were observed in our study. Furthermore, acetate inhibits the release of proinflammatory cytokines (15), as we also observed. Which of the above pathways contributed to the CSY5669 effects remains to be established. To decipher which effects are caused by which signals, control analogs with either a SCFA or a nitro ester should be prepared and investigated.

In summary, we show here that the new AZM analogue CSY5669 stimulates the intracellular killing of MRSA *in vitro* and attenuates detrimental inflammatory responses in the airways of mice with MRSA pneumonia *in vivo* at doses as low as 0.01 μmol/kg p.o. These effects are unlikely to be due to direct antibacterial effects, given the low dose, the intrinsic resistance to AZM, and the sub-MICs observed in tissue. Several investigations in patients have suggested that macrolides can exert beneficial immunomodulatory effects during acute and chronic inflammatory lung diseases (6). The current study highlights the potential of improving AZM immunomodulation by coupling specific signaling molecules to its backbone, presenting CSY5669 as a possible adjunctive therapy in pneumonia caused by MRSA.

## MATERIALS AND METHODS

### Compound synthesis.

CSY5669 was synthesized as described below and provided, along with AZM, by Synovo (Tübingen, Germany): (2S,3R,4S,6R)-4-(dimethylamino)-2-(((2R,3S,4R,5R,8R,10R,11R,12S,13S,14R)-2-ethyl-3,4,10-trihydroxy-13-(((2R,4R,5S,6S)-4-methoxy-4,6-dimethyl-5-(nitrooxy)tetrahydro-2H-pyran-2-yl)oxy)-3,5,6,8,10,12,14-heptamethyl-15-oxo-1-oxa-6-azacyclopentadecan-11-yl)oxy)-6-methyltetrahydro-2H-pyran-3-yl acetate, H NMR (300 MHz, CDCl3, Bruker) δ 7.20 (d, J = 37.8 Hz, 1H), 5.15 (d, J = 4.5 Hz, 1H), 4.80 (d, J = 9.8 Hz, 2H), 4.77 – 4.62 (m, 2H), 4.46 – 4.33 (m, 1H), 4.18 (s, 1H), 3.64 (d, J = 14.9 Hz, 3H), 3.48 (d, J = 6.8 Hz, 1H), 3.31 (s, 3H), 3.21 (d, J = 9.0 Hz, 1H), 2.92 (s, 1H), 2.76 – 2.59 (m, 3H), 2.44 (dd, J = 25.1, 12.9 Hz, 2H), 2.32 – 2.21 (m, 8H), 2.06 (t, J = 12.6 Hz, 1H), 2.00 (s, 3H), 1.90 (s, 3H), 1.79 – 1.57 (m, 3H), 1.43 (s, 2H), 1.23 (d, J = 3.9 Hz, 11H), 1.16 (d, J = 5.2 Hz, 6×H), 1.05 (d, J = 10.4 Hz, 5H), 0.85 (t, J = 7.2 Hz, 8H). 13C NMR (75 MHz, CDCl3, Bruker) δ 178.71, 169.96, 129.07, 128.26, 100.30, 94.55, 87.42, 82.86, 77.86, 77.58, 77.16, 76.74, 74.28, 73.76, 73.45, 71.88, 70.14, 67.75, 63.60, 62.58, 62.42, 49.44, 45.13, 41.85, 40.76, 36.35, 35.41, 30.56, 29.72, 27.47, 26.67, 21.97, 21.58, 21.45, 21.28, 21.17, 17.72, 16.24, 14.69, 11.28, 8.84, 7.50. Purity >99% via HPLC-ELSD. HPLC set up came from Varian (ProStar) and ELS detection (Sedere Sedex 80). Mobile phase: water (0.05% formic acid)/methanol (0.05% formic acid). Stationary-phase: Maisch ReproSil-Pur 120 C18-AQ, 5 μm, 75 × 3 mm).

Azithromycin (17.3 g, 0.023 mol) was transferred into a 500 mL round bottom flask equipped with an addition funnel. To this, glacial acetic acid (138 mL, 2.414 mol) was slowly added, stirring until all solids dissolved. The reaction vessel was cooled in an ice-bath. In another 100 mL round bottom flask, acetic anhydride (68.3 mL, 0.723 mol) was transferred and cooled in an ice bath while nitric acid (7.6 mL, 0.160 mol) was added dropwise. This acetic anhydride/nitric acid solution was transferred to the addition funnel and was introduced to the reaction vessel dropwise (approximately 1 drop/sec). The reaction was stirred for approximately 3 h, and reaction progress was monitored via MS through the disappearance of the starting material or via TLC: 30:1:1 chloroform/2-propranol/7% ammonia in MeOH. The workup was performed by pouring the reaction solution into an ice-water mixture, with constant stirring, until the ice had melted. The solution was neutralized with solid NaHCO_3_ to a pH of at least 8. The aqueous solution was extracted with dichloromethane (3 × 500 mL). The combined DCM layers were washed with saturated aqueous NaCl solution, dried with anhydrous Na_2_SO_4_, and evaporated *in vacuo*, resulting in a mixture of CSY5669 and 4′′-nitrate azithromycin ester. In a second reaction step, crude mixture was dissolved in dichloromethane, and acetic anhydride (17.1 mL, 0.181 mol) was added slowly. The solution was stirred at room temperature for 5 h. Reaction progress was monitored via MS through the disappearance of the starting material or via TLC: 30:1:1 chloroform/2-propranol/7% ammonia in MeOH. The workup was performed by extracting the product with citric acid solution (5%, 3x). Then, the citric acid solution was washed with ethyl acetate. The acidic solution was neutralized with NaHCO_3,_ and macrolide was transferred into organic phase (ethyl acetate) with liquid-liquid extraction three times. Organic phases were combined and washed with brine/water twice, dried with Na_2_SO_4_, and solvent evaporated. Finally, the residue was purified by flash chromatography (Interchim puriFlash 5.020 with Interchim PF-15SIHP-F0040; stationary-phase: silica gel, 2.5 μm, Ø = 3 cm, h = 14 cm; mobile phase: cyclohexane/acetone with triethylamine) to carry out white solid powder (7.1 g, 8,492 mmol).

### Bacterial culture.

S. aureus strain Newman and strain USA300/BK11540 (kindly provided by Timothy J. Foster, Department of Microbiology, Trinity College, Dublin, Ireland) were cultured overnight in liquid phase in Todd-Hewitt broth with yeast extract (THY) until the logarithmic growth phase (i.e., optical density = 1 at 600 nm [OD_600_]). Bacteria were washed twice and either resuspended in a relevant assay medium or heat-killed at 70°C for 30 min and subsequently labeled with 0.4 mg/mL fluorescein isothiocyanate (FITC) (Sigma-Aldrich, Houten, the Netherlands) in 0.1 M NaHCO_3_ (pH 9.0) for 30 min, after which the bacteria were washed and stored at −20°C. Direct antibacterial effects were tested by diluting the bacteria to OD_600_ = 0.1 in THY broth in the presence of AZM or CSY5669 at the indicated concentrations. Two hours after starting the incubation, a sample was taken and serially diluted and plated on blood agar plates to determine bacterial counts. Alternatively, bacteria were incubated overnight at 37°C, and OD_600_ was measured to assess MICs.

### Mouse infection.

Wild-type C57BL/6 mice were purchased from Charles River Laboratories, Inc. (Maastricht, the Netherlands). Mice (*n* = 8 per group) were treated by oral gavage with 0.01 to 10 μmol/kg (i.e., 0.2 to 200 nmol per mouse) CSY5669 dissolved in 100 μL 0.5% citric acid or a vehicle (100 μL of 0.5% citric acid). When all mice were treated, the mice were anesthetized via the inhalation of isoflurane (Abbott Laboratories, Queenborough, UK) and inoculated via intranasal instillation with 50 μL of a saline solution containing a sublethal dose of MRSA (1 × 10^7^ CFU, strain USA300/BK11540) as previously described (20, 43, 44). 6 or 24 h after infection, the mice were sacrificed by cardiac puncture under Domitor (Pfizer Animal Health Care, active ingredient medetomidine) and Nimatek (Eurovet Animal Health, active ingredient ketamine) anesthesia. Blood was collected in heparin tubes. In order to collect bronchoalveolar lavage fluid (BALF), a midline incision was made to expose the trachea, which was cannulated with a 22-gauge Abbocath-T catheter (Abbott Laboratories). Unilateral BALF was collected from the left lung after binding the right bronchus and instilling 0.8 mL of sterile PBS. Organs (i.e., liver, spleen, and right lung) were removed aseptically and split in two. One half was homogenized in four volumes of isotonic saline, while the other half was either collected in 10% formalin for histology (*n* = 4 per treatment group) or snap-frozen for an investigation of pharmacokinetics (*n* = 4). Bacterial loads in homogenized tissues were determined by plating 10-fold dilutions of blood and homogenized tissue on blood agar plates. Colonies were counted after overnight incubation at 37°C.

All animals were housed in the Animal Research Institute Amsterdam under standard care. The experiments were reviewed and approved by the Central Authority for Scientific Procedures on Animals (CCD) and the Animal Welfare Body (IvD) of the Academic Medical Center Amsterdam (approval number: DIX288). The animal care and experimental protocol adhered to the Dutch Experiments on Animals Act (WOD) and the European Directive of 2010 (Directive 2010/63/EU) and 2009 (Directive 2009/41/EC).

### Histology.

Lung histology was determined as previously described (43, 44). Briefly, after the blinding of the samples, all slides were scored by a pathologist on the following parameters: bronchitis, edema, interstitial inflammation, intraalveolar inflammation, pleuritis, endothelialitis, and percentage of the lung surface demonstrating confluent inflammatory infiltrate. Each parameter was graded from 0 to 4, with 0 being “absent” and 4 being “severe”, and the total pathology score was expressed as the sum of all parameters.

### HPLC-MSMS measurements to determine compound concentrations.

CSY5669 and its metabolites were quantified using liquid chromatography coupled to a triple quadrupole MSMS detector (API 4500, Sciex, Canada) as described previously (45). Briefly, samples were homogenized in 6 volumes of acetonitrile, and the resulting supernatant was separated via reverse phase chromatography followed by mass-specific detection. CSY5669, its metabolites, and AZM are well-ionized and stable to this procedure.

### Flow cytometry BALF.

BALF was centrifuged at 250 g for 10 min, after which the supernatant was collected and stored at −20°C for cytokine analyses. The cell pellets were labeled with FITC-labeled anti-mouse Ly-6G, PE-labeled anti-mouse CD3, PE-Fluor610-labeled anti-mouse CD45, PerCP-Cy5.5-labeled anti-mouse CD11c, PE-Cy7-labeled anti-mouse CD11b, AlexaFluor647-labeled anti-mouse Siglec F, AlexaFluor700-labeled anti-mouse Ly-6C (all from BD Biosciences), and efluor780-labeled fixable viability dye (eBioscience). After 30 min of staining, 1-step Fix/Lyse Solution (eBioscience/Thermo Fisher) was added. After 60 min, the cells were washed and resuspended in FACS buffer supplemented with 7000 Precision Counting Beads (BioLegend). The gating strategy is presented in Fig. S1.

### Cytokine analysis.

Tumor necrosis factor (TNF)-α (human and mice), IL-6 (human and mice), IL-10 (human and mice), CXCL1, CXCL2, CXCL5 (all except mouse IL-10: R&D Systems, Minneapolis, MN, USA; mouse IL-10: eBioscience, Thermo Fisher Scientific), elastase, and myeloperoxidase (MPO) (Hycult Biotechnology BV, Uden, the Netherlands) were measured via ELISA according to manufacturers’ recommendations.

### Chemotaxis assay.

Polymorphonuclear neutrophils (PMN) were isolated from venous blood by Ficoll density centrifugation followed by the addition of ice cold erythrocyte lysis buffer (Qiagen) to the cell pellet. After 10 min, the cells were washed twice with ice cold PBS, resuspended in RPMI 1640 supplemented with 10% FCS, and treated with 0 to 10 μM CSY5669. Cells were seeded in a transwell insert, and migration into the “main” well, which contained medium, IL-8 (R&D Systems), or N-formylmethionyl-leucyl-phenylalanine (fMLP, Sigma-Aldrich) was determined after 1 h.

### Cells.

Peripheral blood leukocytes were prepared from human heparinized blood by adding erythrocyte lysis buffer (Qiagen, Hilden, Germany). After 10 min, the cells were washed twice in ice cold PBS and resuspended in RPMI 1640 medium (Life Technologies, Bleiswijk, the Netherlands) supplemented with 10% FBS.

Peripheral blood mononuclear cells (PBMCs) were isolated using Ficoll-Paque density gradient centrifugation from buffy coats derived from blood donation after written informed consent (Sanquin, Amsterdam, The Netherlands). Monocytes isolated with CD14^+^ MACS sorting (MiltenyiBiotec, Bergisch Gladbach, Germany) were differentiated into macrophages (monocyte-derived macrophages) with 5 ng/mL granulocyte-macrophage colony-stimulating factor (GM-CSF; Life Technologies-Invitrogen) as previously reported (46). Cells were cultured in RPMI 1640 (Life Technologies) supplemented with 10% FBS, 2 mM l-glutamine (GlutaMAX; PAA, Linz, Austria), 100 U/mL penicillin, and 100 μg/mL streptomycin (Life Technologies) at 37°C and 5% CO_2_.

All human samples were collected in accordance with the Declaration of Helsinki, after receiving approval from the Academic Medical Center Medical Ethical Committee (approval number: 2015_074), and in agreement with Dutch regulations.

### Bacterial killing assays.

MDMs were infected with bacteria at a multiplicity of infection (MOI) of 10 for 30 min at 37°C and 5% CO_2_. The cells were subsequently washed and treated for 10 min with 30 μg/mL gentamicin (Gibco/ThermoFisher, Leiden, the Netherlands) to kill residual extracellular bacteria. Next, infected cells were incubated at 37°C and 5% CO_2_ for 16 h to allow intracellular killing, after which cells were lysed with ddH_2_O containing 0.05% SDS (5 min up to a maximum of 10 min incubation to prevent any effects on bacterial viability). Cell lysates were serially diluted using broth and plated onto agar plates (blood agar or square Middlebrook TSB-agar). PBLs were treated with AZM (1 μg/mL), CSY5669 (1 μg/mL), or a DMSO vehicle control for 30 min, after which MRSA was added at an MOI of 0.1. If indicated, AZM, CSY5669, or DMSO vehicle were washed off with RPMI 1640 medium before the addition of the MRSA bacteria. After 6 h, cells were lysed with ddH_2_O containing 0.05% SDS, and cell lysates were serially diluted and plated onto blood agar plates.

### Phagocytosis.

FITC-labeled bacteria were opsonized for 30 min at 37°C using 20% fresh human serum diluted in PBS and incubated with MDMs at 37°C and 5% CO_2_ at an MOI of 100. After 60 min, cells were washed with ice cold PBS, resuspended in FACS buffer supplemented with 20% trypan blue (to quench the surface fluorescence of FITC), and measured using flow cytometry (FACSCanto, BD Biosciences).

### Reactive oxygen species (ROS) production and degranulation assay.

To investigate ROS production and neutrophil degranulation, MDMs or PBLs were stimulated with heat-killed MRSA at an MOI of 10 in the presence of 10 μM ROS-reactive dye CellROX green reagent (ThermoFisher) or carboxy-H2DCFDA (Sigma-Aldrich). After 45 min incubation at 37°C and 5% CO_2_, the cells were washed with ice cold PBS and stained for 30 min with either eBioscience Fixable Viability Dye eFluor 780 (ThermoFisher), anti-CD14 PE-Dazzle 594 (BioLegend) and anti-CD11b PE-Cy7 (BD Biosciences), or eBioscience Fixable Viability Dye eFluor 780, anti-CD66b-FITC, anti-PD-L1-PE, anti-CD18-PE-Cy7, anti-CD63-AF647, and anti-CD11b-APC-Cy7, after which cells were washed and measured using flow cytometry (FACSCanto, BD Biosciences). The gating strategies of both assays are presented in Fig. S2.

### Phagosomal maturation.

MDMs were seeded in black poly-d-lysine coated glass 96-well plates (MatTek Corporation, Ashland, MA, USA) and allowed to adhere overnight. MDMs were infected with S. aureus (strain USA300/BK11540) at an MOI of 10 during a 1 h incubation at 37°C and 5% CO_2_. Extracellular bacteria were killed with 30 μg/mL gentamicin for 10 min, after which cells were treated with 1 μM CSY5669 and incubated for 2 h at 37°C and 5% CO_2_. During the last 30 min of incubation, 75 nM Lysotracker Deep Red (ThermoFisher) was added, after which the cells were washed, fixed for 1 h in 1% formaldehyde, washed twice in PBS, and labeled with 2 μg/mL Hoechst 33342 (ThermoFisher) in PBS. After 5 min, the Hoechst 33342 was removed, and ProLong Glass Antifade Mountant was added and cured overnight. Samples were analyzed with a SP8WLL confocal microscope (Leica, Amsterdam, the Netherlands). The Lysotracker channel background was subtracted via the rolling ball algorithm (46 pixel radius) in ImageJ v1.50i (NIH, Bethesda, MD, USA). All images were analyzed using CellProfiler 3.0.0 as previously described (47). In short, GFP-MRSA were segmented by manual global thresholding with intensity-based declumping, and Lysotracker stained objects were segmented by adaptive two-class Otsu thresholding with upper and lower bounds (to minimize bias by cell-specific differences in background signals) as well as with intensity-based declumping. Then, for each image, the percentage of Lysotracker objects whose area overlaps exceeded 50% with individual GFP-MRSA was established, and the average colocalization was calculated for each treatment condition. The numbers of cells and bacteria per image were determined using nuclear Hoechst, and these variables were comparable between different experimental conditions. Three pictures were obtained from each sample, and each experimental condition was performed in triplicate (i.e., 9 pictures per condition, per experiment). Fig. S3 provides additional information on the automated quantification, representative images, and the numbers of bacteria, cells, and Lysotracker identified per image.

### Statistical analysis.

The statistical significance of observed differences was tested using either a (repeated measurement) one-way analysis of variance with Tukey’s *post hoc* test (*in vitro* data) or a Kruskal-Wallis Test with Dunn’s *post hoc* test (*in vivo* data). All tests were two-sided and were performed on nonnormalized data. A *P*-value of <0.05 was considered to be indicative of a statistically significant result. Statistical testing and graphical presentation were performed in GraphPad Prism v8.4.2 (GraphPad Software, San Diego, CA, USA).
