# Supplementary material for: The Azithromycin Pro-Drug CSY5669 Boosts Bacterial Killing While Attenuating Lung Inflammation Associated with Pneumonia Caused by Methicillin-Resistant Staphylococcus aureus
Source: Antimicrob Agents Chemother. 2022 Aug 16;66(9):e02298-21. doi: 10.1128/aac.02298-21 (PMC9487537; doi:10.1128/aac.02298-21)
Supplement: Supplemental file 1 — Fig. S1 to S4. Download aac.02298-21-s0001.pdf, PDF file, 24.8 MB [file aac.02298-21-s0001.pdf]

Supplemental figure 1

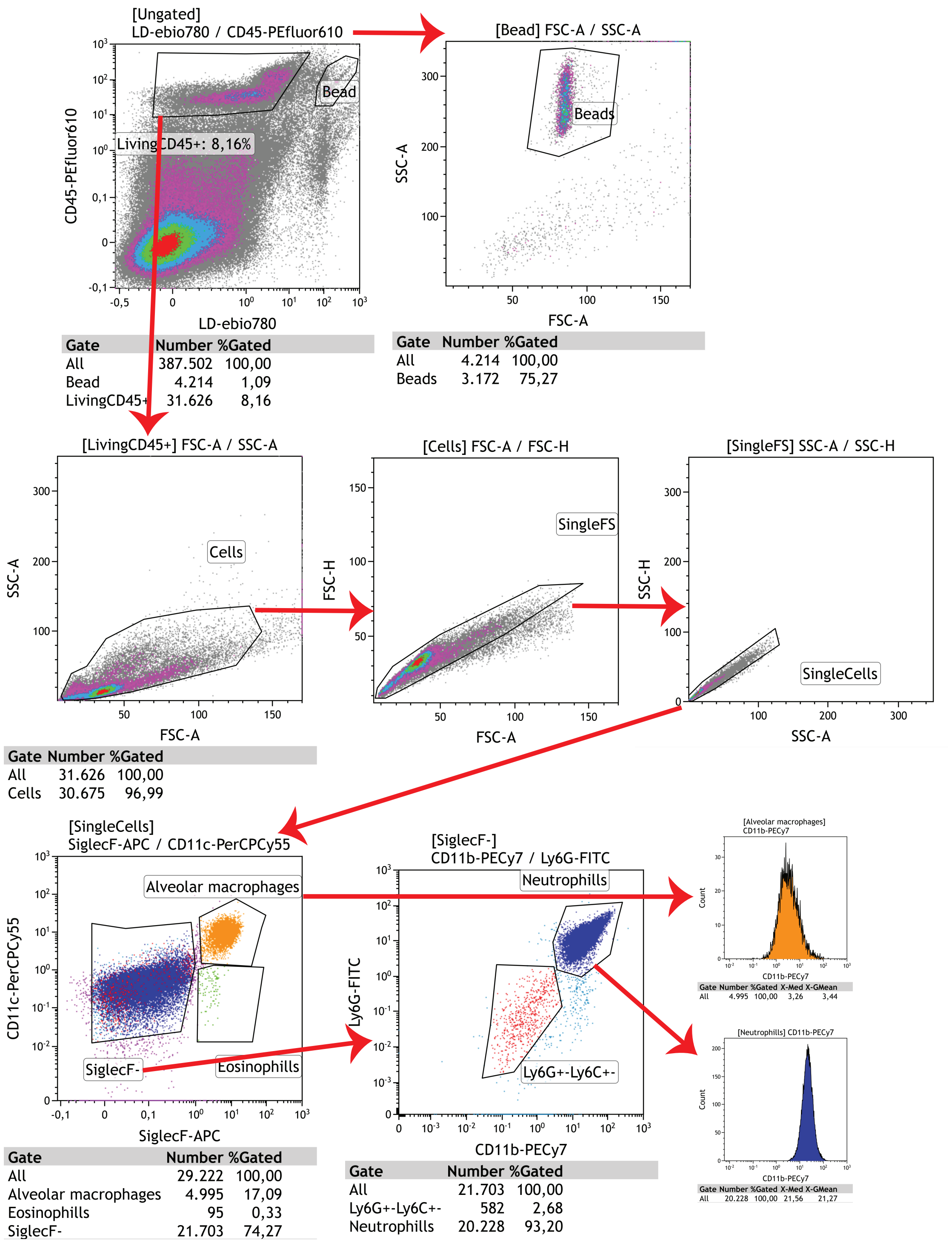

Gate	Number	%Gated
All	387.502	100,00
Bead	4.214	1,09
LivingCD45+	31.626	8,16

Gate	Number	%Gated
All	4.214	100,00
Beads	3.172	75,27

Gate	Number	%Gated
All	31.626	100,00
Cells	30.675	96,99

Gate	Number	%Gated
All	29.222	100,00
Alveolar macrophages	4.995	17,09
Eosinophills	95	0,33
SiglecF-	21.703	74,27

Gate	Number	%Gated
All	21.703	100,00
Ly6G+-Ly6C+-	582	2,68
Neutrophills	20.228	93,20

Gate	Number	%Gated	X-Med	X-GMean
All	4.995	100,00	3,26	3,44

Gate	Number	%Gated	X-Med	X-GMean
All	20.228	100,00	21,56	21,27

Supplemental figure 1: gating strategy as used to determine cell influx into BALF of MRSA-infected mice. Beads were used to quantify absolute cell influx and were fluorescent in all channels.

Supplemental figure 2

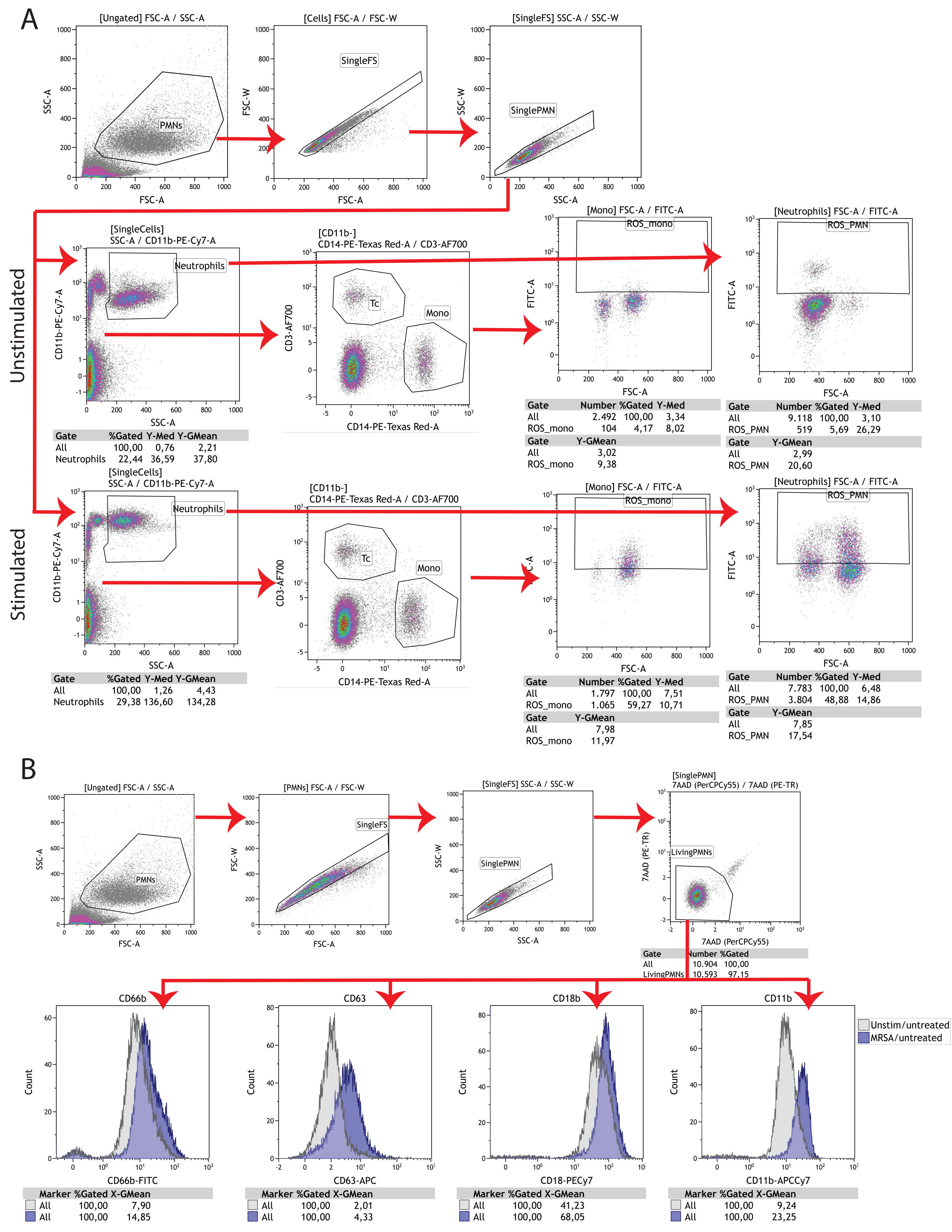

Supplemental figure 2: gating strategies as used in a full blood ROS assay (A) and neutrophil degranulation assay (B). In both unstimulated and MRSA-stimulated samples were included.

Supplemental figure 3

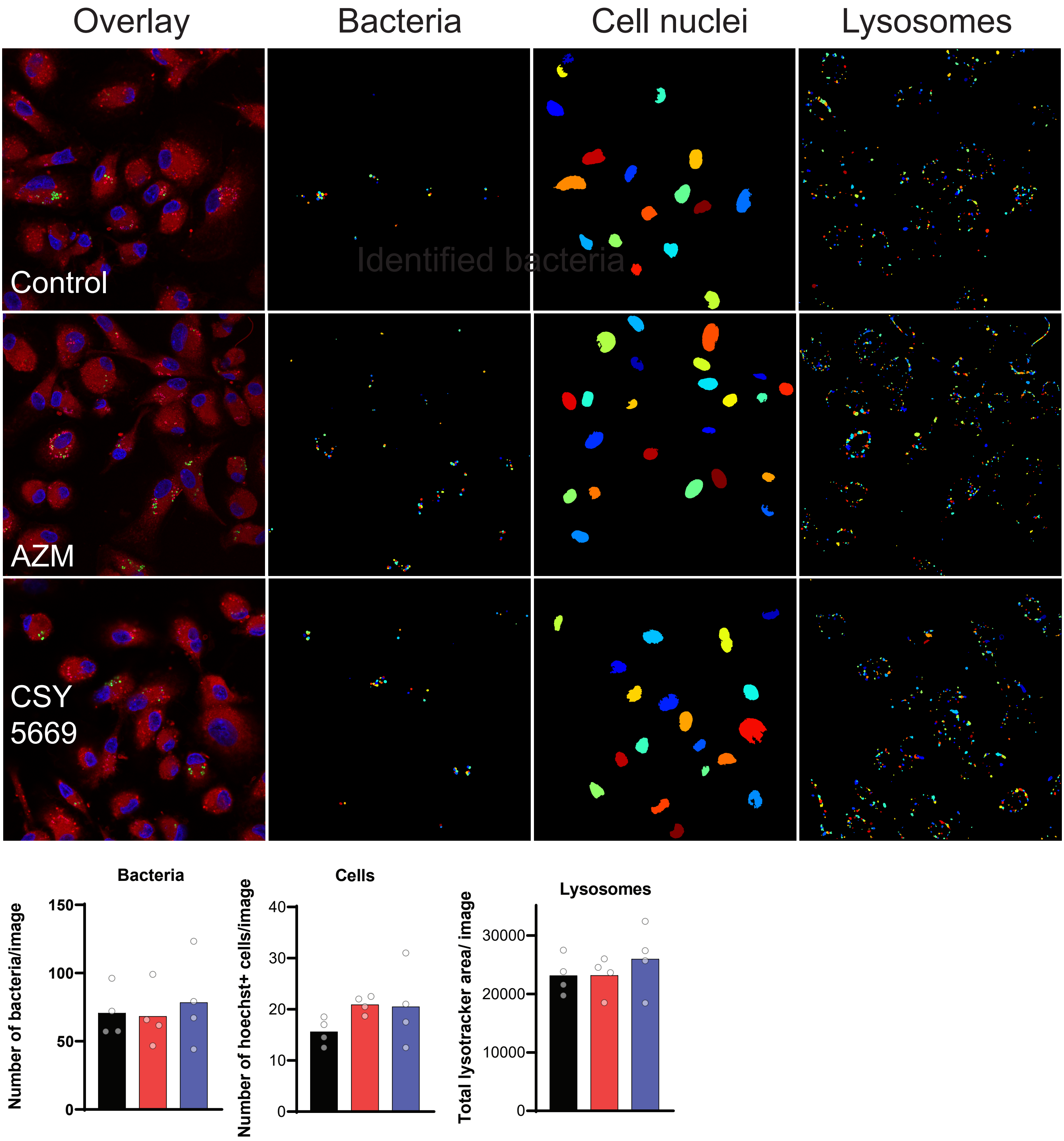

Supplemental figure 3: quantification of bacterial colocalization with lysosomes. Macrophages were infected with GFP-expressing MRSA (green), treated with CSY5669, AZM or control. After 120 min cells cells were stained withy lysotracker (red) and Hoechst nuclear stain (blue) (A). Each condition was performed in triplicate and three images were obtained per sample (i.e. 9 pictures / condition) and presented with one dot in quantification plots. Representative image displaying automated quantification using cellprofiler of bacteria, (cellular) nuclei and lysotracker+ organelles, wherein every individual identified object is presented with a unique color (A). Quantification of number of bacteria, number of cells and total area of lysotracker events is presented. The level of bacterial colocalization and number of lysotracker events identified per cell is presented in Figure 4H.

Supplemental figure 4

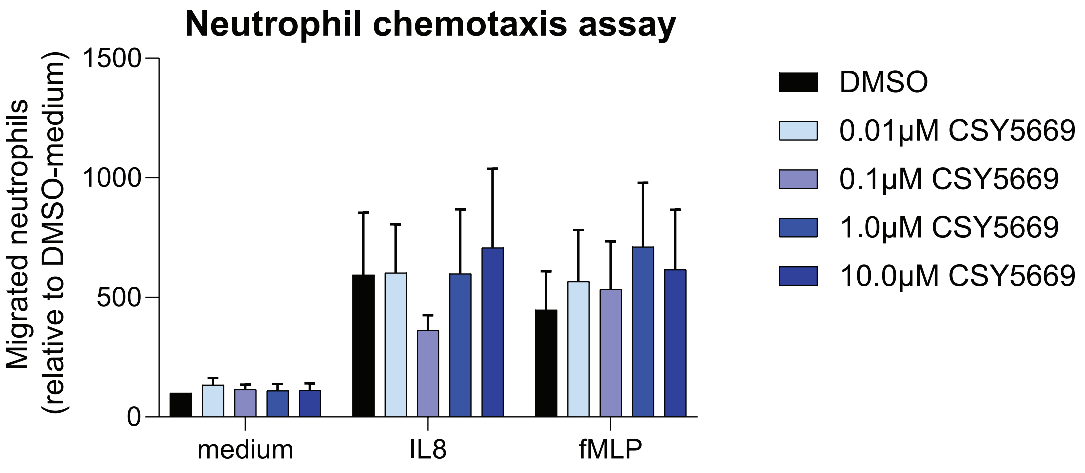

Supplemental figure 4: CSY5669 does not affect neutrophil chemotaxis in vitro. Isolated neutrophils were treated with 0.01-10uM CSY5669 or DMSO and seeded in a transwell insert to determine chemotaxis towards medium, IL-8 or N-formylmethionyl-leucyl-phenylalanine (fMLP) after 1 hour (n=3). Data are presented as mean ± SD. Statistical relevance was tested using one-way ANOVA.
